# The Healing Callus-Promoting Effect of Fenugreek in a Humerus Shaft Fracture: A Case Report

**DOI:** 10.7759/cureus.50519

**Published:** 2023-12-14

**Authors:** Mansour M Aldhilan, Mohamed E Abdel-Wanis

**Affiliations:** 1 Orthopedic Surgery, Al Rass General Hospital/Ministry of Health, Ar Rass, SAU

**Keywords:** bony callus, case report, callus formation, fracture healing, trigonella, fenugreek seed

## Abstract

A 54-year-old male presented with a fractured shaft in the right humerus and refused surgery. The patient was treated with a cast, and a follow-up plain radiography revealed good callus formation after 32 days. The patient had a history of receiving fenugreek seed extract from the first week after the fracture. We did our best to exclude any other factors that helped rapid fracture healing with good callus formation in our patient. The current case supports the hypothesis that fenugreek seed extract promotes bone healing. This hypothesis is supported by a literature review. Previous studies have suggested several mechanisms by which fenugreek promotes bone healing.

## Introduction

Bone fracture management aims to heal a fracture in a good anatomical position within a reasonable time frame. Two factors would affect fracture healing: mechanical and biological [1,2]. *Trigonella foenum-graecum*, often known as fenugreek, is an annual plant with an extended history of use as a traditional medicine [3]. Fenugreek seeds are useful for treating and preventing different ailments [4]. Flavonoids, such as apigenin 6,8-di-C-glucoside, apigenin-6-C-glucosyl-8-C-galactoside, 6-Cgalactosyl- 8-C-arabinoside, as well as rhaponticin and isovitexin, are the main components of fenugreek seeds. Fenugreek seeds contain phosphorus, which falls into different classes: inorganic phosphorus, phospholipids, phytates, phosphor-proteins, and nucleic acids. Germinated seeds contain amino acids, proteins, ascorbic acid, vitamin E, and sugars [4-7]. A previous study showed that mandibular fracture healing had been accelerated in camels fed fenugreek (100 g/camel/day) for two weeks [3]. However, to our knowledge, no previous study has reported the beneficial effects of fenugreek seeds on fracture healing in humans. This study aimed to report a case in which fenugreek seed extract enhanced the healing of a fracture. 

## Case presentation

**Figure 1:**
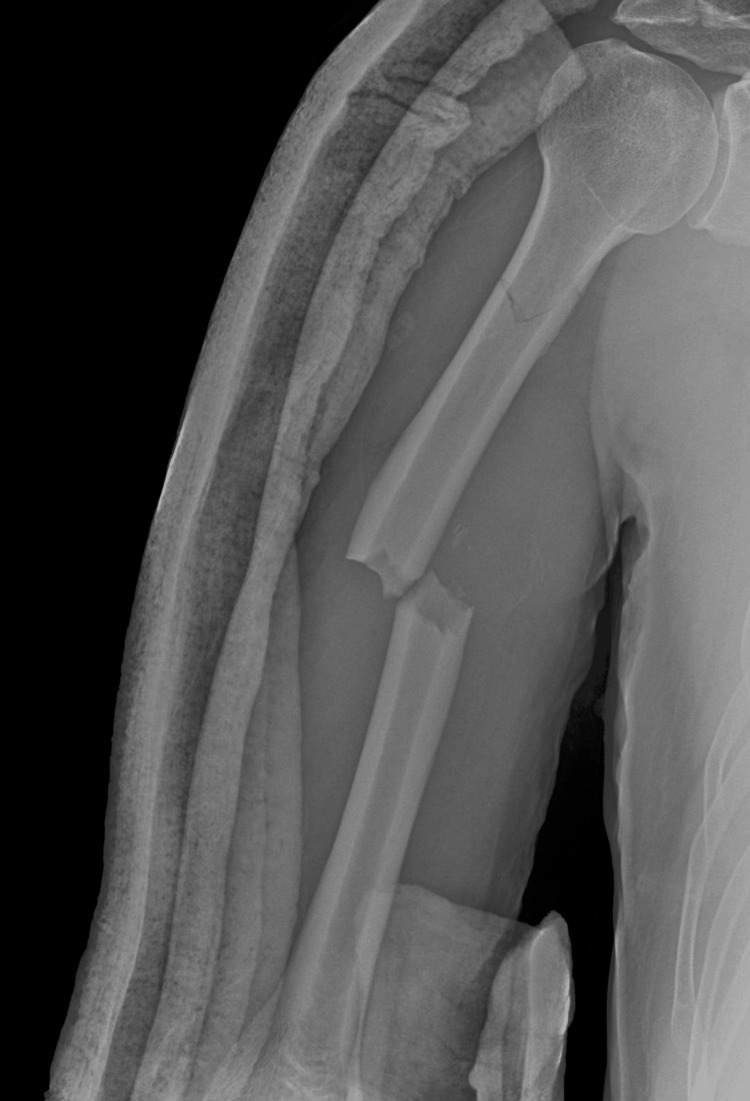
Plain radiograph shows right humerus fracture on the day of the accident

A 54-year-old male had a road traffic accident on May 10, 2023. Patient history showed that the patient was medically free, with no past surgical or medical history, but with chronic sinusitis. The patient weighed 75 kg, had a height of 156 cm, and had a body mass index (BMI) of 30.82. The patient had no history of head trauma or loss of consciousness. Plain radiograph showed a right mid-shaft humerus fracture and a proximal fissure fracture (Figure 1).

**Figure 2:**
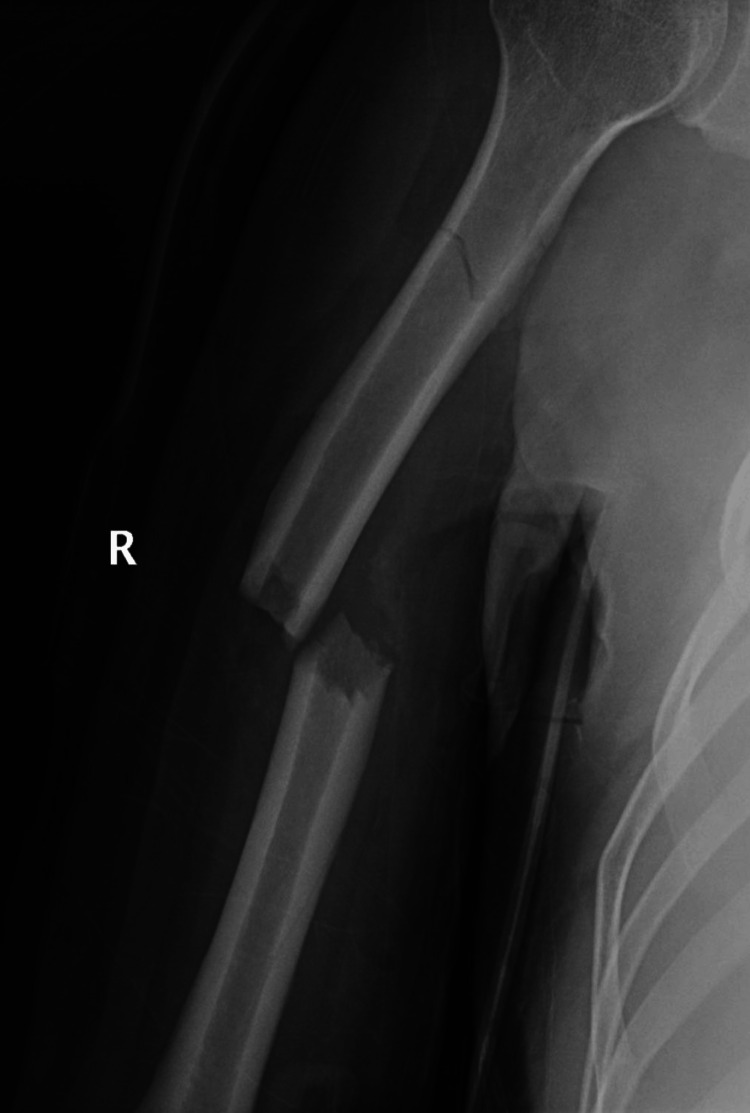
Plain radiograph shows a right humerus fracture callus starts to appears 11 days after the accident

Computed tomography (CT) of the brain, abdomen, and pelvis was unremarkable. CT of the chest showed posterior lung field ground-glass opacity, likely contusion. Laboratory findings showed a normal renal panel and the random blood sugar level was 148 mg/dL (normal 80-140 mg/dL). Complete blood analysis showed a white blood cell count to be 14.8 (normal 4-10) x 10^3^, hemoglobin 14.9 (normal 13-17) g/dL, and platelet count 270 (normal 150-400) x 10^3^ U/L. The patient refused surgical intervention and was asked to undergo conservative treatment with a cast on the injured arm. On follow-up, the plain radiograph on May 21, 2023 (11 days after the fracture) showed that a callus had started to appear (Figure 2).

**Figure 3:**
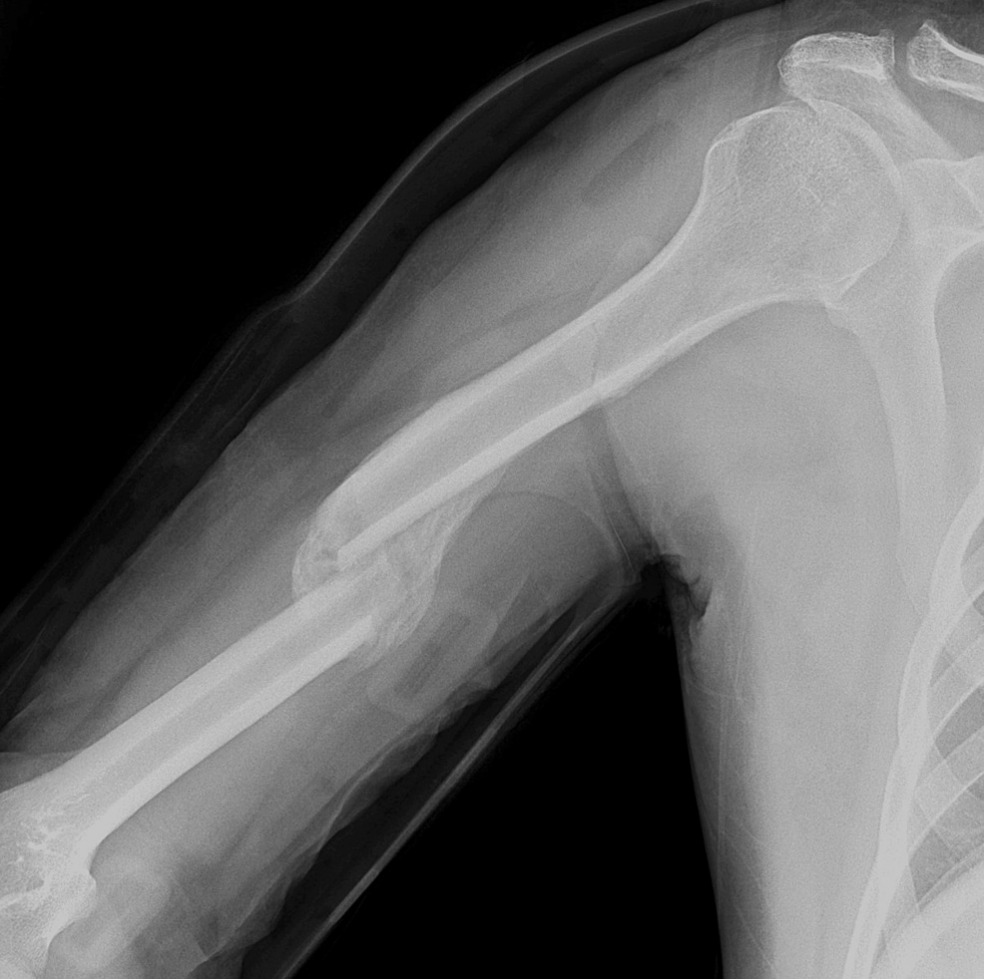
Plain radiograph shows a right humerus callus 32 days after the accident

On follow-up, a plain radiograph on Jun 11, 2023 (32 days after fracture) showed a large callus formation (Figure 3).

The patient's history confirmed regular consumption of fenugreek aqueous seed extract two to three times a day starting from the first week of the fracture.

## Discussion

Previous studies have suggested several mechanisms by which fenugreek promotes bone healing. It controls osteogenic markers and increases osteoblast differentiation by raising calcium levels outside of cells and promoting osteoblast differentiation. Experimental studies have shown that it increases osteocalcin and bone morphogenic protein-2 (BMP-2) levels in treated cells. It inhibits tartrate-resistant acid phosphatase (TRAP), prevents proliferation, inhibits invasion, suppresses osteoclastogenesis via inhibition of receptor activator of nuclear factor kappa beta (RANKL/NF-kappaB)-regulated gene expression, exhibits significant DNA damage to osteoclast cells, and enhances apoptosis [6,8]. It contains free amino acids, such as histidine [5] and calcium [9], both of which are important in callus formation and fracture healing [1,2]. Alkaline phosphatase (ALP) participates in the biological processes of fracture healing. Fenugreek stimulates ALP activity in bone marrow-derived stem cells [8,10].

Fenugreek has antihyperglycemic characteristics of type I and type II diabetes in both humans and animals. Research conducted in diabetic rats given alloxan demonstrates that dialyzed fenugreek seed extract exhibits hypoglycemic activity similar to insulin. [5,11]. Gupta et al. reported the results of a double-blind placebo study of patients with type II diabetes mellitus recently diagnosed. The results of their study proved that using fenugreek seeds enhances glucose regulation and decreases insulin resistance [12,13]. Dyslipidemia, characterized by elevated total and low-density lipoprotein cholesterol concentrations, is primarily linked to low bone mass and an increased risk of fracture. Elevated oxidative stress and systemic inflammation linked to dyslipidemia directly cause this impact, raising osteoclastic activity and decreasing bone production. Elevated cholesterol inhibits bone growth and hinders osteoblast development. Therefore, enhanced osteoclastogenesis might be involved in this process. Low high-density lipoprotein C (HDL-C) concentrations have been linked to an inflammatory microenvironment's formation and elevated bone marrow adiposity, which restricts osteoblast differentiation and function and reduces bone mass [14,15]. Experimental studies have shown that fenugreek significantly reduces triglyceride (TG) levels and causes a significant increase in HDL-C levels [13,16]. 

Different experimental studies have shown that oxidative stress affects osteoblasts, osteoclasts, and osteocytes, causing an imbalance between bone formation and resorption in favor of bone resorption as well as impairing bone mineralization [17-19]. Several studies have suggested fenugreek as a potential antioxidant. Bhatia et al. examined lipid peroxidation and antioxidants in the urine bladders of mice given cyclophosphamide, demonstrating the protective effects of fenugreek on enzymatic antioxidants and lipid peroxidation [20]. 

Fenugreek's callus-promoting effect in humans is suggested for the first time in this reported case. We did our best to exclude any other factors that helped rapid fracture healing with good callus formation in our patient. No head trauma occurred in our patient. The patient did not receive any biophysical enhancement of fracture healing, such as electromagnetic field or low-intensity pulsed ultrasonography. No systemic therapies, such as teriparatide, calcium, or vitamin D, which might accelerate fracture healing, were given to the patient. [2]. Some side effects were reported after the use of fenugreek, like dyspepsia, abdominal distension, hypersensitivity reactions, cross-reactivity in favism, hypoglycemia, and the risk of bleeding due to the coumarin content. Drinking fenugreek extract in patients receiving some medication should be done with caution as it potentiates the effect of the anticoagulant drug and may reduce potassium serum levels. Additionally, it may increase the hypoglycemic effect of antidiabetic treatment. Its use during pregnancy is not recommended as it may stimulate uterine contractions [9].

This case report has some limitations, and we retrospectively reviewed our case. We could not confirm the recommended dose or duration of fenugreek. In addition, our patient was not assessed for calcium level, vitamin D, or biological markers of bone healing, including serum ALP and osteocalcin. We realize that the evidence level of our case is low; however, it would suggest the need for higher-level evidence from well-planned randomized controlled trials (RCT) that include follow-up of fracture healing using serial radiographs as well as serum bone formation and resorption biomarkers.

## Conclusions

This case report suggests the callus-promoting effect of fenugreek seed extract in patients with fractures. However, well-planned randomized controlled trials are required to confirm this effect. Moreover, verification is required for the safe dosage and duration of fenugreek usage. Nevertheless, we were not confronted with any side effects of fenugreek in our patient.
